# Gene expression profiles of breast biopsies from healthy women identify a group with claudin-low features

**DOI:** 10.1186/1755-8794-4-77

**Published:** 2011-11-01

**Authors:** Vilde D Haakensen, Ole Christian Lingjærde, Torben Lüders, Margit Riis, Aleix Prat, Melissa A Troester, Marit M Holmen, Jan Ole Frantzen, Linda Romundstad, Dina Navjord, Ida K Bukholm, Tom B Johannesen, Charles M Perou, Giske Ursin, Vessela N Kristensen, Anne-Lise Børresen-Dale, Åslaug Helland

**Affiliations:** 1 Dept of Genetics, Institute for Cancer Research, Oslo University Hospital Radiumhospitalet, 0310 Oslo, Norway; 2 Institute for Clinical Medicine, Faculty of Medicine, University of Oslo, 0316 Oslo, Norway; 3 Dept of Oncology, Oslo University Hospital Radiumhospitalet, 0310 Oslo, Norway; 4 Biomedical Research Group, Department of Informatics, University of Oslo, 0316 Oslo, Norway; 5 Center for Cancer Biomedicine, University of Oslo, 0310 Oslo, Norway; 6 Department of Clinical Molecular Biology, Division of Medicine and Laboratory Sciences, Institute for Clinical Medicine, Akershus University Hospital, University of Oslo, 0316 Oslo, Norway; 7 Dept of Surgery, Akerhus University Hospital, 1478 Lørenskog, Norway; 8 Dept of Epidemiology and Lineberger Comprehensive Cancer Center, University of North Carolina at Chapel Hill, Chapel Hill, NC 27599-7264, USA; 9 Lineberger Comprehensive Cancer Center, University of North Carolina at Chapel Hill, Chapel Hill, NC 27599-7264, USA; 10 Dept of Radiology, Oslo University Hospital Radiumhospitalet, 0310 Oslo, Norway; 11 Dept of Radiology, University Hospital of North Norway, 9038 Tromsø, Norway; 12 Dept of Radiology, Buskerud Hospital, 3004 Drammen, Norway; 13 Dept of Radiology, Innlandet Hospital, 2609 Lillehammer, Norway; 14 Institute of Health Promotion, Akershus University Hospital, 1478 Lørenskog, Norway; 15 The Norwegian Cancer Registry, 0304 Oslo, Norway; 16 Dept of Nutrition, School of Medicine, 0316 University of Oslo, Oslo, Norway; 17 Dept of Preventive Medicine, University of Southern California Keck School of Medicine, Los Angeles, CA 90089, USA

**Keywords:** Gene expression, normal breast tissue, hierarchical clustering, claudin-low

## Abstract

**Background:**

Increased understanding of the variability in normal breast biology will enable us to identify mechanisms of breast cancer initiation and the origin of different subtypes, and to better predict breast cancer risk.

**Methods:**

Gene expression patterns in breast biopsies from 79 healthy women referred to breast diagnostic centers in Norway were explored by unsupervised hierarchical clustering and supervised analyses, such as gene set enrichment analysis and gene ontology analysis and comparison with previously published genelists and independent datasets.

**Results:**

Unsupervised hierarchical clustering identified two separate clusters of normal breast tissue based on gene-expression profiling, regardless of clustering algorithm and gene filtering used. Comparison of the expression profile of the two clusters with several published gene lists describing breast cells revealed that the samples in cluster 1 share characteristics with stromal cells and stem cells, and to a certain degree with mesenchymal cells and myoepithelial cells. The samples in cluster 1 also share many features with the newly identified claudin-low breast cancer intrinsic subtype, which also shows characteristics of stromal and stem cells. More women belonging to cluster 1 have a family history of breast cancer and there is a slight overrepresentation of nulliparous women in cluster 1. Similar findings were seen in a separate dataset consisting of histologically normal tissue from both breasts harboring breast cancer and from mammoplasty reductions.

**Conclusion:**

This is the first study to explore the variability of gene expression patterns in whole biopsies from normal breasts and identified distinct subtypes of normal breast tissue. Further studies are needed to determine the specific cell contribution to the variation in the biology of normal breasts, how the clusters identified relate to breast cancer risk and their possible link to the origin of the different molecular subtypes of breast cancer.

## Background

Early diagnosis of breast cancer is essential for reducing both mortality and morbidity of the disease. Knowledge of the initial steps of breast carcinogenesis is important for development of early detection strategies. Breast carcinogenesis, with the transition of normal breast epithelial cells through hyperplasia to invasive cancer, is increasingly well understood [1,2], but there is uncertainty as to the exact mechanisms of tumour initiation and in which cells these first steps occur [3]. In order to obtain a better understanding of breast cancer biology, breast carcinogenesis and origin of the different molecular subtypes of breast cancer, information about normal breast biology and its variability among women is essential.

In breast carcinomas, the variability of gene expression has been extensively studied. Several expression subtypes have been identified [4,5]. These subtypes are partly believed to originate from different cell types of the breast, the luminal subtypes from luminal epithelial cells and the basal-like subtype from a myoepithelial or a possible luminal progenitor cell type [6]. Recently, an additional subtype has been identified [7], the claudin-low subtype, which, based on gene its expression profile, is characterized by low expression of luminal markers and high expression of mesenchymal markers. This subtype is associated with bad prognosis and is thought to be derived from stem cells [8].

The normal breast consists of epithelial cells, extracellular matrix with stromal cells, adipose tissue and breast stem cells that reside in the stem cell niche [9]. The stem cell niche prevents the epithelial stem cells from differentiating and is defined by stroma [10]. Epithelial breast cells may be of luminal or myoepithelial type and they may undergo epithelial-mesenchymal transition and gain mesenchymal characteristics. Several groups have published lists of genes characterising these various cell-types [11-19].

Whole genome expression profiling of normal breast tissue (all cell types included) from women with no malignant disease has been performed only to a limited extent so far, in studies with other aims and with few samples [5,20-22]. In this study we explore the expression profiles of normal breast tissue from a series of healthy women and to what extent they varied across demographic data such as age, body mass index, hormone therapy use and parity. The expression profiles obtained mirror the combined gene activity of the different cell types in the biopsy, reflecting a fingerprint of the breast tissue of that particular woman. Analyzing normal breast tissue from healthy women may identify biological significant subtypes of normal breast tissue. This could be of importance for understanding the different expression patterns seen in the various breast cancer subclasses [4,5].

## Methods

### Materials

#### MDG - mammographic density and genetics

The mammographic density and genetics (MDG) project was initiated to study the breast biology of healthy women and in particular the biological/genetic basis for mammographic density. Women included in the study were recruited from several mammographic centers in Norway between 2002 and 2007 as previously described [23]. Most women were referred to the mammographic center after some irregular or questionable findings in an initial mammogram. A total of 120 women who were evaluated as cancer-free from routine diagnostic procedures were included in the study. Women with some visible areas of mammographic density were included in order to obtain biopsies from these areas with epithelial and stromal components. If there was a suspicious lesion in one breast, the study biopsy was taken from the breast contralateral to the lesion. A total of 66 women with newly diagnosed breast cancer were included for comparison and biopsies were taken from the tumor. Women who used anticoagulants, had breast implants, were pregnant or breast feeding were excluded. All women signed an informed consent and answered a questionnaire with information about parity, family history of breast cancer and hormone use. Breast biopsies and blood samples were collected. The hospital research protocol board and the regional ethical committee (ref: S-02036) approved the study. Data from the questionnaires was stored in a database organized by the Office for Clinical Research at the Oslo University Hospital; Radiumhospitalet.

Core biopsies for the study were obtained using a 14 gauge needle. The biopsies were taken from an area without pathology but with some mammographic density. Six healthy women included by one hospital, had the biopsy taken from a non-malignant lesion (five from fibroadenomas and one from a microcalcification). The biopsies from one hospital (66 healthy women) were fresh frozen and stored at -80°C. The remaining hospitals placed the biopsies directly on RNAlater (Applied Biosystems/Ambion, Austin, TX) before transportation and storage at -80°C.

Mammographic density was estimated using the University of Southern California Madena assessment method [24] as previously described [23]. Briefly, the total breast area was outlined by an operator. The area containing densities and excluding the pectoralis muscle and artifacts was marked and the threshold set to select the densities within this area. Percent density is the dense area divided by the total breast area and was used as a measure of mammographic density. Information about which of the included subjects that had developed breast cancer by April 2010 was collected from the Norwegian Cancer Registry.

#### Other datasets

Gene expression profiles from breast biopsies of healthy women included in the MDG study were compared with one published and two unpublished gene expression datasets. The two unpublished dataset were from the Akershus University Hospital (AHUS). The 40 AHUS1-samples were histologically normal tissue collected from two different cohorts; 26 breasts harboring breast cancer (hereafter called cancer normals) and 14 mammoplasty reductions. The 13 AHUS2-samples were collected from different sources selected to different proportion of fatty and connective tissues. Breast tissue was sampled from mammoplasty reductions, fibroadenomas and normal tissue from breast cancer mastectomies. In addition, subcutaneous fat was collected from the abdominal area. The samples were grouped into biopsies with high and low fraction of fat tissue based on visual inspection. In both AHUS datasets, RNA was extracted from whole tissue. In addition, one published dataset containing reduction mammoplasties and cancer normals was used [20].

Previously published lists of genes differentially expressed between epithelial cells and stem-like/progenitor cells, stromal cells, myoepithelial cells or epithelial cells after epithelial-mesenchymal transition were used to describe our dataset. The genes from each publication are listed in Supplementary file 1.

### Gene expression analysis

RNA-extraction and hybridization to microarrays were done as previously described [23]. Briefly, RNeasy Mini Protocol (Qiagen, Valencia, CA) was used for RNA-extraction. Agilent Low RNA input Fluorescent Linear Amplification Kit Protocol was used for cDNA-synthesis, transcription and labeling of RNA with cyanine 5 (Amersham Biosciences, Little Chalfont, England) for the samples and cyanine 3 (Amersham Biosciences, Little Chalfont, England) for the Universal Human total RNA reference (Stratagene, La Jolla, CA). After exclusion of 38 samples due to low amount of RNA or poor RNA-quality, 82 samples were hybridized onto two-channel 44K Agilent Human Whole Genome Oligo Microarrays (G4110A) (Agilent Technologies, Santa Clara, CA). Three arrays were excluded due to poor quality, and 79 samples from healthy women were included in further analyses and are available in Gene Expression Omnibus (GEO) GSE18672. From the breast cancer cases, 64 gene expression experiments were included for further analysis.

### Data processing

An Agilent scanner (Agilent Technologies, Santa Clara, CA) was used for scanning and Feature Extraction 9.1.3.1 (Agilent Technologies, Santa Clara, CA) was used for data processing. Normalization was done by locally weighted scatterplot smoothing (lowess) and flagged spots were removed. The Stanford Microarray Database (SMD) [http://genome-www5.stanford.edu//] was used for data storage. For further analysis the log 2 transformed data were used. The genes were filtered so that only genes with 80% good data and a log2-value of more than 1.6 standard deviation away from the mean in three samples or more were included leaving 9767 probes. A gene filtering using genes with a log2-value of more than 1.2 was also tested, without significant alterations of the results.The data were gene-centered for cluster analysis, but not for other analyses. Missing values were imputed in R using the method impute.knn in the library impute [http://rss.acs.unt.edu/Rdoc/library/impute/html/impute.knn.html]. The AHUS1-dataset was filtered to include the probes in the filtered MDG-dataset, leaving 8519 probes.

The data were checked for effect of handling (fresh frozen versus RNAlater) and batch using significance testing, Envisage [http://www2.warwick.ac.uk/fac/sci/moac/currentstudents/2003/sam_robson/linear_models/] and visualization by multidimensional scaling and single value decomposition. Samples with questionable array quality were re-run. The conclusion was that a slight effect of batch and date of hybridization using uncorrected data is seen, but this did not affect the clustering. Fisher exact and chi-squared tests were used to analyze for difference between our two main clusters using batch, experiment date, storage medium, RNA-concentration and hospital of inclusion as variables. The results of these were all negative showing no effect of sample handling or collection site. Also, there was no correlation between sampling method/storage medium and RNA-amount or quality.

### Statistical Analysis

Clustering was performed using MatLab (version R2007b) (The MathWorks Inc., Natick, MA) with ward linkage and Euclidean distance measure. Average linkage and Pearson correlation coefficient were also tested without significant alterations of the results. The gap statistic was used to determine the number of clusters [25]. Two-sided t-tests (assuming equal variance) and chi-squared/Fisher's exact tests were used to test for statistical significant differences in phenotypic variables between clusters. Significance Analysis of Microarrays (SAM) (version 3.02) [http://www-stat.stanford.edu/~tibs/SAM/] [26] for Excel with 500 permutations was used for analysis of differentially expressed genes. The empirical null distribution was estimated to ensure that the genes identified as differentially expressed between the two clusters did not merely represent the tails of a wider null distribution. Unsupervised hierarchical clustering was performed using the complete gene list filtered as described above. Supervised analyses were performed using different published gene lists to look for similarities between the different cell types from which the respective gene sets were derived.

Prediction of the claudin-low subtype was done using the claudin-low predictor developed in Prat et al [27]. An expression dataset with 807 genes and 52 cell line samples (described in Neve et al, [28]), of which 9 were classified as claudin-low, was merged with our data using Distance Weighted Discrimination [29] with the 52-sample dataset used as the training data. In the same software, the single sample prediction (SSP) function with Euclidean distance was applied on the adjusted datasets and then used to define claudin-low samples in the test set.

A similar predictor was developed for prediction of the previously identified intrinsic subtypes. A dataset containing both the original intrinsic subtypes and the claudin-low subtype [7] was merged with our data set as described above for the cell line data. The Herschkowitz-dataset was used as the training data. The single sample prediction was applied to assign expression subtypes to the samples.

Subtypes of the samples that were not called claudin-low were estimated by PAM50 with the -1 option in calibration parameters [30]. A dataset containing gene expression values of the 79 normal breast biopsies and the 64 breast cancer biopsies was used. The genes were filtered to include the genes used for subtyping by PAM50. The genes of all samples were centered by subtracting the mean gene expression in tumor samples only.

Microsoft Access 2003 was used to limit our dataset to the gene lists of interest. The gene lists used are listed in Additional file 1. Hierarchical clustering was performed to see whether the gene list of interest separated the cluster 1-samples from the remaining samples in our dataset. SAM of cluster 1 versus cluster 2 was performed to identify genes from the published gene list that were differentially expressed between cluster 1 and cluster 2. Tests for significance between the number of up- and down-regulated genes (false discovery rate (FDR)<10%) between the two clusters identified and the cell types in question were performed. Gene set enrichment analysis (GSEA) (version 2) [http://www.broadinstitute.org/gsea/] with 1000 permutations was used to check for significance of the gene lists in separating the clusters. DAVID 6.7 8 [http://david.abcc.ncifcrf.gov/home.jsp] was used to identify gene ontology terms and KEGG pathways significantly enriched in the gene lists differentially expressed between the two main clusters. Terms with an FDR<0.01 were considered statistically significant.

Clustering combining the MDG dataset with datasets containing biopsies from normal tissue containing different proportions of adipose tissue was performed to see whether samples in cluster 1 consistently clustered with samples with a high fraction of fat tissue and was driven by a high number of adipocytes.

## Results

### Unsupervised hierarchical clustering

**Figure 1:**
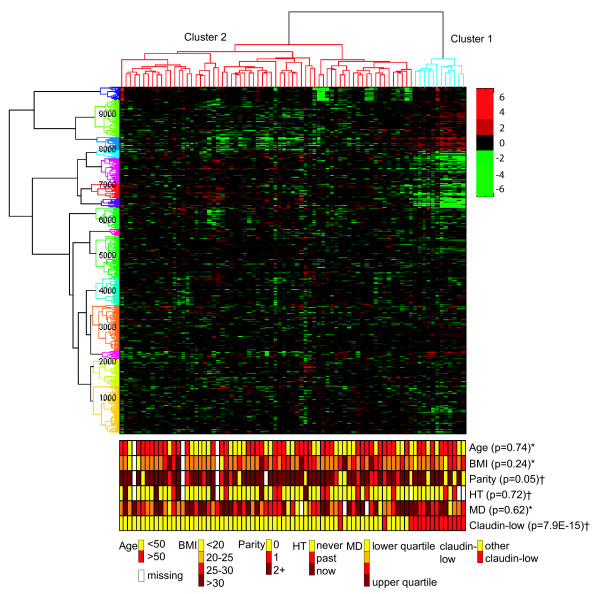
**Unsupervised hierarchical clustering of 79 samples from healthy individuals and 9767 genes filtered on variation**. Phenotypes with tests for significant difference in values between cluster 1 (blue) and cluster 2 (red). Continuous variables are categorized for the illustration, but significance tested as continuous variables. P-values from two-sided t-tests assuming equal variance for continuous variables (*) and chi-squared tests (**) for categorical variables are given. The numbers along the y-axis denotes the number of genes. Age = Age at time of inclusion. BMI: Body mass index. HT: Use of hormone therapy. MD: Mammographic density.

Unsupervised hierarchical clustering of the expression of 9767 genes in the 79 breast biopsies separated the samples into two main groups (Figure 1). This was also confirmed by the gap statistic [25]. The smaller cluster (cluster 1, far right), consisting of twelve samples, consistently clustered tightly together regardless of clustering method and gene filtering. There was a significantly higher proportion of women referred to mammography due to increased risk (family history of breast cancer (n = 4) or a palpable breast lump (n = 5)) in cluster 1 compared to the remaining women (cluster 2). No malignancy was found in any of the women included in the study by standard diagnostic procedures. Women in cluster 1 were slightly more likely to be nulliparous, compared to cluster 2 (p = 0.05). They were also more likely to be referred from a doctor due to increased risk rather than from the screening program, compared to cluster 2 (p = 0.002). There was no difference in age, age at first birth, hormone use, body mass index or percent mammographic density between women belonging to the two clusters (Table 1).

**Table 1:** Women included in the study, descriptive statistics

		all non-cancer (%)	cluster 1 (%)	cluster 2 (%)	p-value (cluster 1 vs 2)
Age	mean	50.2	49.3	49.8.4	0.87 ^1)^
	<40	14 (18)	3 (25)	11 (16)	
	40-49	17 (22)	2 (17)	15 (22)	
	50-69	43 (54)	6 (50)	37 (55)	
	70+	2 (3)	1 (8)	1 (1)	
	missing	3 (4)	0 (0)	3 (4)	

Parity	0	10 (13)	4 (33)	6 (9)	**0.05**^2)^
	1+	65 (82)	8 (67)	57 (85)	
	missing	4 (5)	0 (0)	4 (6)	

Age at first birth	mean	24.4	24	24.6	0.75 ^1)^
	no children	10 (13)	4 (33)	6 (9)	
	<25	30 (38)	4 (33)	26 (39)	
	25-34	24 (30)	3 (25)	21 (31)	
	35+	2 (3)	0 (0)	2 (3)	
	missing	13 (16)	1 (8)	12 (18)	

Hormone therapy use	never	55 (70)	8 (67)	47 (70)	0.72 ^2)^
	current	11 (14)	2 (17)	9 (13)	
	past	6 (8)	0 (0)	6 (9)	
	missing	7 (9)	2 (17)	5 (7)	

Body mass index	mean	24	23	24.5	0.24 ^1)^
	<20	5 (6)	2 (17)	3 (4)	
	20-<25	44 (56)	6 (50)	38 (57)	
	25-<30	21 (27)	4 (33)	17 (25)	
	30+	6 (8)	0 (0)	6 (9)	
	missing	3 (4)	0 (0)	3 (4)	

Mammographic density	mean	39.6	36.7	37	0.62 ^1)^
	0-<23	19 (24)	2 (17)	17 (25)	
	23-<37	21 (27)	4 (33)	17 (25)	
	37-<52	19 ((24)	3 (25)	16 (24)	
	52+	17 (22)	3 (25)	14 (21)	
	missing	3 (4)	0 (0)	3 (4)	

Referral source	risk/lump	32 (41)	9(75)	23 (33)	**0.007**^2)^
	screening	28 (36)	0 (0)	28 (42)	
	unknown	19 (24)	3 (25)	16 (24)	

1) Two-sided t-test for continuous variables

2) Fisher exact test for categorical variables with <5 observations in certain cells

### Differentially expressed genes

SAM revealed 2621 genes differentially expressed between cluster 1 and cluster 2 with an FDR = 0, of which 1516 were up-regulated in cluster 1 (Additional file 2).

**Figure 2:**
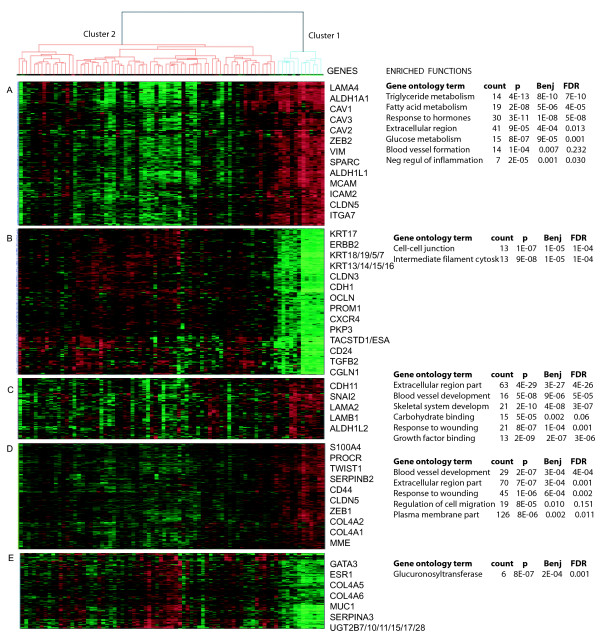
**Selected genes from gene clusters up- and down-regulated in cluster 1**. Gene functions/ontology terms associated with the respective gene clusters are given. In cluster 1 there is an up-regulation of mesenchymal genes and stem-cell related genes (A, C and D) and down-regulation of epithelial markers and claudins (B and E).

Genes up-regulated in cluster 1 were enriched for the gene ontology terms extracellular region, vascular development, response to hormone stimulus, glucose and triclyceride metabolism, plasma membrane, cell motion and regulation of inflammatory response. Genes down-regulated in cluster 1 were enriched for the terms of proteins involved in actin-binding, adherens junction, cytoskeleton and the plasma membrane (Additional file 3, Table S1). Gene ontology terms associated with subsets of genes in the various gene clusters (A-E) are shown in Figure 2.

### Supervised analyses

In order to explore the nature of the cells in the biopsies of cluster 1, we used previously published gene lists describing stroma [17,18], breast stem cells [15,19,31], myoepithelial cells [12,14], progenitor cells [14], mesenchymal cells [13], high-risk normal cells [16], epithelial cells from parous women [32], intrinsic genelist [5] and a genelist for prediction of the claudin-low subtype [27].

Both hierarchical clustering, SAM analysis and GSEA indicated that the expression in the cluster 1-biopsies resembled expression in stem-like cells and stromal cells (Table 2) (Additional file 3, Table S2). There were also certain shared expression characteristics with progenitor cells, mesenchymal cells and myoepithelial cells. More detailed information about the cells used when generating the gene lists, the samples used and the number of genes from the respective gene lists differentially expressed in our clusters are listed in Additional file 3, Table S3. The cluster 1 samples were not associated with the expression profiles of any of the original breast cancer subtypes [5]. However, when a gene list developed to classify the newly identified claudin-low subtype was used [27], we found that the cluster 1 samples were highly associated with the claudin-low gene expression profile (Table 2). This was confirmed when we used this method to create a predictor for one subtype at a time. All samples in cluster 1 were classified as claudin-low as opposed to only three samples from cluster 2 (Figure 1) and the cluster 1 samples were not assigned to any of the other subtypes tested using these predictors. In Figure 2, selected genes associated with the claudin-low subtype, stem cells, mesenchymal cells, stroma and epithelial cells and their expression in cluster 1 are shown. Hierarchical clustering based on the various gene lists is shown in Additional file 3, Figure S1. These analyses could not confirm any association of cluster 1 with parity [32].

**Table 2:** Comparison of cluster 1 and cell types/subtypes from published gene lists

Comparison	Reference	Cluster 1 resembles	P-value
Epithelial vs stem-like cell	Shipitsin, 2007	Stem-like cell	**2.20E-16**
Stroma vs epithelium	Finak, 2006	Stroma	**2.20E-16**
Mesenchymal vs epithelial	Jechlinger, 2003	Mesenchymal	**6.90E-14**
Revised subtypes	Herschkowitz, 2007	Claudin-low	**1.52E-12**
Fibroblasts vs epithelial cells	Casey, 2008	Fibroblasts	**1.9E-12**
Risk predictor	Chen, 2009	Low risk	**1.30E-09**
Stem-like cell vs epithelial	Liu, 2007	Stem-like	**1.30E-05**
Myoepithelial vs progenitor	Raouf, 2008	Progenitor	**2.40E-05**
Luminal vs progenitor	Raouf, 2008	Progenitor	**0.001**
Stem-like vs progenitor cells	Villadsen, 2007	Lineage restricted progenitor	**0.008**
Myoepithelial vs luminal	Jones, 2004	(Myepithelial)	0.06
Classical subtypes	Sorlie, 2001	-	0.76

*) Chi squared test for significance

Chi-squared test is used to illustrate the extent to which genes describing different cell types are equally regulated in the two clusters. Significant p-values are in bold type. For more information on the publications and comparisons, see Supplemental file 3, Table S3.

When the filtered expression dataset was clustered with three separate datasets including biopsies from breasts of healthy women with high and low content of fatty tissue (two unpublished AHUS-datasets and one published [20] dataset), the samples did not cluster according to fat-content (Additional file 3, Figure S2).

Four of the women from the MDG-study have been registered with a breast cancer diagnosis, all in the breast contralateral to the biopsy. The samples from these four women all belonged to cluster 2. The observation time varied from 34 to 86 months with a mean of 59.1 and a median of 58. All four cancers were estrogen receptor positive. Two of the breast cancers developed after inclusion were infiltrating lobular carcinoma, and two had ductal histology.

**Figure 3:**
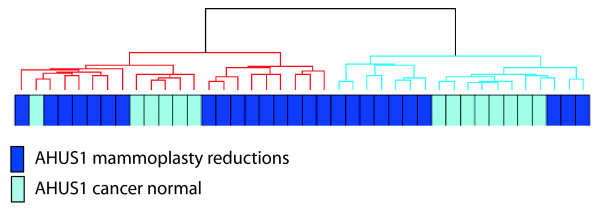
**Unsupervised hierarchical clustering of 40 samples from the validation dataset AHUS1**. Reduction mammoplasties and cancer normal samples are split between one larger and one smaller cluster, the smaller cluster containing slightly more mammoplasty reductions.

Unsupervised hierarchical clustering of the AHUS1-dataset yielded two distinct clusters of samples (Figure 3). The smaller cluster (n = 18) includes 8 reduction mammoplasties, while the larger cluster (n = 22) included 6. A total of 3102 of 8519 probes were differentially expressed between the two clusters using SAM. Of the 2045 genes up-regulated in the smaller cluster with an FDR of 2%, 1057 were also up-regulated in cluster 1 in the MDG dataset whereas none were down-regulated. Of the 2278 genes down-regulated in the smaller cluster with and FDR of 2%, 962 were down-regulated in cluster 1 and none were up-regulated.

Gene ontology terms enriched in the genes up- regulated in the smaller AHUS1-cluster were very similar to those found in the cluster 1-samples, including: glucose and triglyceride metabolism, vasculature development, response to hormone stimulus, regulation of lipid metabolic process, sulfur metabolic process and membrane cell fraction. In addition, the gene ontology terms mitochondrion and vitamin B6-binding, propanoate metabolism and cellular respiration were enriched in the genes up-regulated in the smaller cluster of AHUS1. The genes down-regulated in this smaller cluster were enriched for cell junction, cell-cell junction and extracellular matrix - the majority of terms being in common with those down-regulated in the cluster 1 samples. The full list of gene ontology terms for both datasets is available in Additional file 3, Table S1.

Application of the claudin-low predictor on the AHUS1-samples showed that no samples in the larger cluster were assigned claudin-low as opposed to 16 of 18 in the smaller cluster where most samples were called normal-like (suppl file 3, figure S3)

## Discussion

Little is known about gene expression patterns in normal breasts. We have identified a cluster of twelve normal breast tissue samples (cluster 1) that cluster tightly together using different clustering algorithms and different gene lists and that share characteristics of stromal cells, stem cells and the claudin-low phenotype.

The cluster 1 samples have a reduced expression of the epithelial defining keratin genes and have an up-regulation of several mesenchymal markers such as *TWIST1*, *SPARC *and *VIM*. This may lead to the hypothesis that the cluster 1 samples represent more immature or dedifferentiated epithelial cells, and/or enrichment for stromal cells. This is supported by our findings that the cluster 1 samples have an expression of genes that resembles published gene lists characterizing stromal tissue and have an overrepresentation of gene ontology terms associated with the extracellular matrix. Reliable and specific stem cell markers are still unavailable [33], but cells in the cluster 1 samples show similarities with stem-like or progenitor-like cells.

The interindividual differences observed may reflect true differences between women with different risk or exposure histories, or may represent different normal tissue subtypes that are present within a single woman, at different sites in the breast, at different times during the lifespan or in different proportions. For example, stem cell niches may be oversampled in the cluster 1 biopsies.

The stem cell niche refers to a zone of the breast epithelium where stem and progenitor cells reside. The microenvironment constitutes the niche and influences the stem cells [34](for review, see [35]). Stem cell niches are thought to be present in the breasts of all women, but some women may have more than others. The immature breasts of nulliparous women may contain larger volumes of stem cell niches than the post-lactationally involuted breasts. This could explain why there are more nulliparous women in cluster 1 than in cluster 2. Understanding the intra- and inter-individual variation in normal breast tissue is important and this investigation raises the question as to whether the clustering patterns observed represent only a fraction of women or if all women have cells/niches with these characteristics, with some women having a higher fraction than others.

The fact that the stem cell niche is constituted by the microenvironment could explain the combined stem-like and stromal-like characteristics identified in cluster 1-samples. In breast cancer, the stem cell niche may contain mesenchymal cells derived from the normal breast stroma or recruited from the bone marrow [10] and the current results raise the hypothesis that mesenchymal cells may be present in normal breast stem cell-niches. The link between mesenchymal and stem cell traits is also made clear by Mani and colleagues who showed that immortalized breast cells undergoing epithelial-mesenchymal transition acquire stem-cell like characteristics and that normal mouse mammary stem cells express mesenchymal markers [36].

This study is not designed to predict risk of developing breast cancer. However, we do have four separate sources of information that can be used to infer about the risk of developing breast cancer: Mammographic density, source of referral to the breast diagnostic center, occurrence of breast cancer after inclusion in the study and the previously published malignancy risk predictor developed by Chen and colleagues [16]. The information from these sources does not point in the same direction. There is no difference in mammographic density, one of the strongest risk factors for breast cancer, between the two clusters (Table 1). When we apply the malignancy risk predictor the cluster 1-samples tend to have a slightly decreased risk. The women with samples in cluster 1 with known referral patterns were all referred to the mammographic centers due to palpable breast lumps or positive family history and not from the screening program. All the four breast cancers developed in these women after inclusion in the study occurred in women belonging to cluster 2, and none in cluster 1. This is not statistically significant due to low numbers. All four cancers were estrogen receptor positive.

The malignancy risk predictor is dominated by proliferative genes and may represent proliferation more than risk of developing breast cancer. Low proliferation rate is also seen in stem cells and an increased proportion of stem cells may explain the low proliferation estimated. The increased incidence of family history of breast cancer in the women belonging to cluster 1 could point toward a higher risk of developing breast cancers by genetic as opposed to environmental causes. The four cancers diagnosed in women belonging to cluster 2 were all estrogen receptor positive, supporting a more environmental/hormonal etiology. Cluster 1 is smaller than cluster 2 and the lack of cancers in this cluster is not statistically significant. The two clusters may not be different in risk of breast cancer as much as in which type of breast cancer the women are predisposed to develop. Since the cluster 1-samples have a stem-like gene expression profile, have certain myoepithelial/basal characteristics and a higher frequency of family history of breast cancer, one may speculate that these women, if they develop breast cancer, will have a greater proportion of estrogen receptor negative cancers

All the 12 samples in cluster 1 were classified as claudin-low, compared to only three of the remaining 67 samples. Similarly in the AHUS1-dataset, the claudin-low samples were exclusively in the smaller cluster which is the one resembling the MDG cluster 1. The claudin-low subtype is developed for classification of breast cancers and was not thought to be a group of normal breast samples. The claudin-low nature of the cluster 1 samples is, however, striking. Down-regulation of E-cadherin, occludin, claudin 3, 4, 7 as well as up-regulation of the mesenchymal genes and SNAI2 is in line with the features described in claudin-low tumor samples. The low expression of ESR1 corresponds with the estrogen receptor negative trend of the claudin-low subtype [7]. The claudin-low tumours are thought to arise from mammary stem cells [8]. The hypothesis that the cluster 1-samples are enriched for immature cells is further supported by the down-regulation of GATA3 seen in these samples compared to the cluster 2 samples (p = 3.8E-9), a protein that is also down-regulated in claudin-low samples [27].

The biopsies used in this study are unique in that they represent a group of women that are examined at breast diagnostic centers. Since the sample size is small, the use of additional datasets is important for validation of the results. The AHUS1 dataset consists of two main types of samples; mammoplasty reductions and cancer normals. Mammoplasty reductions are widely used as representing normal breast tissue, although one can expect the biology to be slightly biased toward fat-related processes. Cancer normals may be influenced by the biology in the cancer [37] or they may represent normal tissue in high-risk breasts [38]. A dataset consisting of these two tissue-types, therefore represent a variety of normal tissue. The fact that the AHUS1 dataset clusters into two clusters with biology similar to those seen in the MDG dataset is interesting and indicates that our results are reproducible.

The reduced expression of epithelial surface makers may be explained by a large component of adipocytes in the biopsies. This is, however, unlikely, as the biopsies were taken from mammographic dense areas. In addition, when this dataset was clustered with other datasets containing biopsies from normal breast tissue with varying proportions of fatty tissue, the cluster 1-samples did not segregate with the adipocyte-rich biopsies (Additional file 3, Figure S2).

There was a greater proportion of nulliparous women in cluster 1. The association between cluster and parity was, however, not confirmed using a gene list describing post-pregnant epithelial cells [32](Table 2 and Additional file 3, Table S3 and Figure S1). The breasts of nulliparous women are not fully matured and the fraction of differentiated epithelial cells is lower than in post-pregnant breasts. The genelist published by Asztalos et al is short and may not capture all parity-related gene expression alterations. The cluster 1-samples may represent more immature breasts with less differentiated epithelial cells, but the association between the cluster 1-type gene expression profile and parity needs to be elucidated further.

The difference between cluster 1 samples and the remaining normal samples could be due to differences in fractions of the cell types present in the biopsies. For ethical reasons, the number of biopsies per woman was limited and we did not have enough tissue to do both RNA-extraction and obtain histology. The lack of histology of the biopsies prior to extraction prevents exact knowledge of the cell types contributing to the expression profiles. It has become evident that the development and progression of breast cancers are not limited to epithelial cells and that the total microenvironment is important. Approximately 95% of normal breast tissue may be composed of stroma, and therefore cell type differences in stroma are most likely captured rather than subtle differences in epithelial content. For evaluation of the putative interplay between all the cells at this location of the breast, expression analysis of the entire biopsy provides the most comprehensive picture of the situation. Previous studies have shown that different biopsies from one tumor share gene expression profile [4]. The variability of gene expression from different locations of one breast is not known, but King and colleagues have shown that microdissected and bulk tissue samples from normal breasts have a high similarity in gene expression and that such technical differences are minor compared with biological differences [39]. There is, therefore, reason to believe that the variability seen represents differences that affect the biology of the breast and not only random sampling.

This study is limited by the relatively low number of women included. Larger datasets with several biopsies representing different parts of the breast will be needed to allow further study of the variation in the normal biology of the breast.

The similarity of the cluster 1 gene expression profile with stromal and stem-like gene signatures and less prominent with mesenchymal cells suggest a biology dominated by less developed cells. This is further supported by the trend of more nulliparous women and the striking similarity with the claudin-low breast cancer phenotype. These samples may represent breasts with an increased number of non-proliferating and not differentiated stem cells with the accompanying stromal niche. There seem to be fewer differentiated luminal cells. We hypothesize that the women belonging to cluster 1 have an increased risk of claudin-low and basal like breast cancer. This is supported by the immature and partly myoepithelial features of the breast and the increase of positive family history in this group.

## Conclusion

Gene expression analyses of biopsies from breasts of healthy women show two main groups of expression patterns. The samples of the smaller group of biopsies cluster tightly together independent of clustering algorithm and gene filtering used. These samples share characteristics with stromal cells and stem cells and are all classified as claudin-low. These findings are reproduced in a separate dataset of normal breast tissue. Whether these characteristics represent traits of the woman or cell niches present in all breasts is unknown. This cluster may represent the stem cell niche, defined by stromal tissue and containing stem-like cells. There are more nulliparous women in this cluster. The described signature may be a feature more prominent of the immature breasts of nulliparous women. We cannot conclude about breast cancer risk between the two clusters, although we see an overrepresentation of women a positive family history of breast cancer or a palpable breast lump in the smaller cluster. We hypothesize that the cluster 1-samples represent breasts with more immature and undifferentiated cells, including stem cells and the accompanying stromal niche and that women belonging to cluster 1 have an increased risk of developing claudin-low or basal-like breast cancer. Further studies are needed to verify the hypotheses generated by this pilot study.

## List of abbreviations

SAM: significance analysis of microarrays; FDR: false discovery rate; MDG: Mammographic density and genetics; AHUS: Akershus University Hospital.

## Competing interests

The authors declare that they have no competing interests.

## Authors' contributions

VDH assisted in data collection, contributed to the lab work, did statistical analyses of the data, interpreted the results and wrote the paper. OCL did statistical analyses of the data. TL contributed to the laboratory work. AP assisted in statistical analysis of the data. MAT assisted in data collection and statistical analysis of the data. MR, MMH, JOF, LR, DN, IKB, TBJ and CMP assisted in data collection. GU designed the trial and estimated mammographic density. VNK designed the trial. ALBD designed the trial and interpreted the results. ÅH designed the trial, assisted in data collection, interpreted the results and wrote the paper. All authors were involved in reviewing the report. No medical writers were involved in this paper. All authors have read and approved the final manuscript.

## Pre-publication history

The pre-publication history for this paper can be accessed here:

http://www.biomedcentral.com/1755-8794/4/77/prepub

## Supplementary Material

Additional file 1 **Genelists from the literature**. Genelists obtained from different publications describing various breast cells. Reference to each publication and listings of Agilent IDs and gene symbol for all genes included in each list. Click here for file

Additional file 2 **Significanc analysis of microarrays (SAM) of cluster 1 vs 2**. A file showing input and output from SAM analysis including list of differentially expressed genes with Agilent IDs, gene symbol, scores, fold change and q-values. Click here for file

Additional file 3 **Supplementary material**. **Table S1: **Gene ontology terms enriched in the genes differentially expressed between the two main clusters in the MDG dataset (cluster 1 and 2 in Figure 1) and the AHUS1 dataset (small and large cluster in Figure 2). **Table S2: S2 **Gene set enrichment analysis (GSEA) of cluster 1 versus cluster 2 using selected gene lists from the literature. **Table S3: **Comparison of cluster 1 and cell types/subtypes from published gene lists. The number of genes up- and down-regulated is given for genes characterizing each cell type and each cluster. **Figure S1: **Hierarchical clustering of gene expression of 79 samples from breasts of healthy women. The samples are clustered based on gene lists from the literature, describing different cell types. **Figure S2: **Biopsies from healthy women (MDG) clustered with two unpublished datasets including mammoplasty reductions and tumor adjacent normal breast tissue. **Figure S3: **Unsupervised hierarchical clustering of 79 samples from breasts of healthy women. Includes estimated subtype of non-claudin-low samples. Click here for file
