# Supplementary material for: Gene expression profiles of breast biopsies from healthy women identify a group with claudin-low features
Source: BMC Med Genomics. 2011 Nov 1;4:77. doi: 10.1186/1755-8794-4-77 (PMC3216859; doi:10.1186/1755-8794-4-77)
Supplement: Additional file 3 — Supplementary material. Table S1: Gene ontology terms enriched in the genes differentially expressed between the two main clusters in the MDG dataset (cluster 1 and 2 in Figure 1) and the AHUS1 dataset (small and large cluster in Figure 2). Table S2: S2 Gene set enrichment analysis (GSEA) of cluster 1 versus cluster 2 using selected gene lists from the literature. Table S3: Comparison of cluster 1 and cell types/subtypes from published gene lists. The number of genes up- and down-regulated is given for genes characterizing each cell type and each cluster. Figure S1: Hierarchical clustering of gene expression of 79 samples from breasts of healthy women. The samples are clustered based on gene lists from the literature, describing different cell types. Figure S2: Biopsies from healthy women (MDG) clustered with two unpublished datasets including mammoplasty reductions and tumor adjacent normal breast tissue. Figure S3: Unsupervised hierarchical clustering of 79 samples from breasts of healthy women. Includes estimated subtype of non-claudin-low samples. [file 1755-8794-4-77-S3.PDF]

Supplemental material

Table S1 Gene ontology terms enriched in the genes differentially expressed between the two main clusters in the MDG dataset (cluster 1 and 2 in Figure 1) and the AHUS1 dataset (small and large cluster in Figure 2).

	Cluster 1 (MDG)			Smaller cluster (AHUS1)		
	**Term**	**Count**	**FDR**	**Term**	**Count**	**FDR**
up	extracellular region part	115	1.6E-10	mitochondrion	179	2.5E-16
	vasculature development	44	6.4E-08	generation of precursor metabolites and energy	62	1.4E-07
	glucose metabolic process	32	5.2E-07	glucose metabolic process	38	1.8E-06
	extracellular matrix part	28	7.9E-07	response to hormone stimulus	66	2.2E-06
	response to hormone stimulus	52	2.4E-06	carboxylic acid biosynthetic process	37	9.7E-06
	regulation of lipid metabolic process	25	1.7E-05	triglyceride metabolic process	18	2.5E-05
	triglyceride metabolic process	15	7.9E-05	regulation of lipid metabolic process	28	3.5E-04
	cell fraction (membrane)	106	1.8E-04	regulation of lipid catabolic process	12	0.004
	response to wounding	58	0.002	cell fraction (membrane)	134	0.006
	plasma membrane part	178	0.004	Propanoate metabolism	14	0.007
	regulation of cell migration	26	0.013	vasculature development	43	0.009
	regulation of response to external stimulus	25	0.014	sulfur metabolic process	25	0.024
	positive regulation of fatty acid metabolic process	9	0.017	vitamin B6 binding	16	0.027
	cell motion	51	0.020	regulation of lipid storage	10	0.032
	blood circulation	27	0.025	positive regulation of fatty acid metabolic process	10	0.032
	response to extracellular stimulus	30	0.026	cellular respiration	22	0.046
	extracellular matrix organization	19	0.029			
	sulfur metabolic process	20	0.034			
	response to steroid hormone stimulus	27	0.044			
down	cell junction	41	0.007	cell junction	52	5.8E-4
	cell-cell junction	22	0.007	cell-cell junction	26	0.004
	plasma membrane part	117	0.012	extracellular matrix	36	0.021

Table S2 Gene set enrichment analysis (GSEA) of cluster 1 versus cluster 2 using selected gene lists from the literature. Significant FDR-values are marked in bold.

Gene list	FDR	Cluster 1
STROMA (FINAK)	**0.00**	Up
STROMA (CASEY)	**0.02**	Up
CD44+ (SHIPITSIN)	**0.01**	Up
BIPOLAR (VS LUM) (RAOUF)	0.10	Up
BIPOTENT (VS MYO) (RAOUF)	0.08	Up
MYOEPITHELIAL (JONES)	0.13	Up
IGS/CD44+ (LIU)	0.20	Up
EPITHIAL (FINAK)	**0.00**	Down
CD24+ (VS CD44) (SHIPITSIN)	**0.03**	Down
IGS/CD10 (LIU)	0.07	Down
RISK (CHEN)	0.24	Down
LUMINAL (JONES)	0.48	Down
EPITHIAL (JECHLINGER)	0.70	Down
MESENCHYMAL (JECHLINGER)	0.66	Down

Table S3 Comparison of cluster 1 and cell types/subtypes from published gene lists. The number of genes up- and down-regulated is given for genes characterizing each cell type and each cluster. Chi-squared test is used to illustrate the extent to which genes describing different cell types are equally regulated in the two clusters. The right column shows samples correctly identified by hierarchical clustering of the normal breast samples based on the gene list from the corresponding publication (see Supplemental file 1, Figure S2)

Publication	Cell type	Method	up in cluster 1	down in cluster 1	cluster 1 resembles	Samples correctly identified by clustering	
						Cluster 1	Cluster 2
Shipitsin, 2007	Epithelial	CD24+	132	152	CD44+	12/12	66/67
	Stem cell-like	CD44+	394	89			
	Unchanged		0	106			
	Sum		526	347			
	$\chi 2$	p-value	**2.2E-16**				
Jechlinger, 2003	Mesenchymal	Before and after TGFbeta-induced EMT	61	17	Mesen-chymal	11/12	67/67
	Epithelial		38	32			
	Unchanged		0	36			
	Sum		99	85			
	$\chi 2$	p-value	**6.9E-14**				

Table S3 cont

Publication	Cell type	Method	up in cluster 1	down in cluster 1	cluster 1 resembles	Samples correctly identified by clustering	
						Cluster 1	Cluster 2
Raouf, 2008	Luminal	CD49flow/ MUC1high/ CD10low	521	442	Progenitor	12/12	67/67
	Bipotent progenitor	CD49fhigh/ MUC1low/ CD10high	259	162			
	Unchanged		343	338			
	Sum		1123	942			
	$\chi 2$	p-value	**0.001**				
Raouf, 2008	Myoepithelial	CD49flow/ MUC1low/ CD10high	139	88	Progenitor	12/12	67/67
	Bipotent progenitor	CD49fhigh/ MUC1low/ CD10high	232	128			
	Unchanged		156	171			
	Sum		527	387			
	$\chi 2$	p-value	**2.40E-05**				
Liu, 2007	Stem cell-like	CD44+	50	17	Stem-like Invasive cells	11/12	67/67
	Epithelial	CD10+	34	58			
	Unchanged		36	27			
	Sum		120	102			
	$\chi 2$	p-value	**1.30E-05**				
Jones, 2004	Myoepithelial	MUC1+	65	91	-	12/12	66/67
	Luminal	CD10+	21	47			
	Unchanged		30	28			
	Sum		116	166			
	$\chi 2$	p-value	0.06				
Chen, 2009	IDC-like normal	Based on gene expression	11	81	Low risk	11/12	62/67
	Other normal c		17	4			
	Unchanged		0	0			
	Sum		28	85			
	$\chi 2$	p-value	**1.3E-09**				
Finak, 2006	Stroma	Micro-dissection	457	91	Stroma	12/12	67/67
	Epithelial		51	317			
	Unchanged		59	92			
	Sum		567	500			
	$\chi 2$	p-value	**2.2E-16**				

Table S3 cont

Publication	Cell type	Method	up in cluster 1	down in cluster 1	cluster 1 resembles	Samples correctly identified by clustering: Cluster 1	Samples correctly identified by clustering: Cluster 2
Casey, 2008	Fibroblasts	Micro-dissection	330	140	Fibroblasts	11/12	64/67
	Epithelial		119	134			
	Unchanged		224	228			
	Sum		673	502			
	χ2	p-value	**1.9E-12**				
Villadsen, 2007	Stem-like	K19+/ K14+	71	104	Lineage restricted progenitor K19+/K14-	7/12	29/67
	Lineage restricted progenitors	K19+/ K14-	46	30			
		K19-/K14+	4	5			
		K19-/K14-	48	81			
	Unchanged		0	0			
	Sum		169	220			
	χ2	p-value	**0.008**				
Asztalos, 2010	Nullipara	Micro-dissection	8	2	Post-pregnant		
	Postpregnant		5	4			
	Unchanged		0	0			
	Sum		13	6			
	Fisher exact	p-value	0.35				
Sørlie, 2001	Basal-like	Based on gene expression	62	66	-	12/12	67/67
	HER2-enriched		72	69			
	Luminal A		73	80			
	Luminal B		70	60			
	Normal-like		80	91			
	Unchanged		0	0			
	Sum		357	366			
	χ2	p-value	0.76				
Hersch-kowitz, 2007	Basal-like	Based on gene expression	56	79	claudin-low	12/12	67/67
	Claudin-low		121	33			
	HER2-enriched		76	98			
	Luminal		70	95			
	Normal-like		76	67			
	Unchanged		163	116			
	Sum		562	488			
	χ2	p-value	**1.52E-12**				

Figure S1

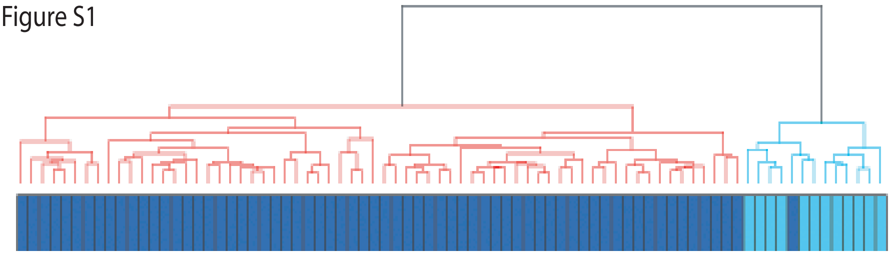

Shiptisin et al, 2007
Luminal (CD24+) vs
stem-cell like (CD44+) cells
χ^2 p-valdue: 2.2E-16
Bonferroni: 2.8E-15

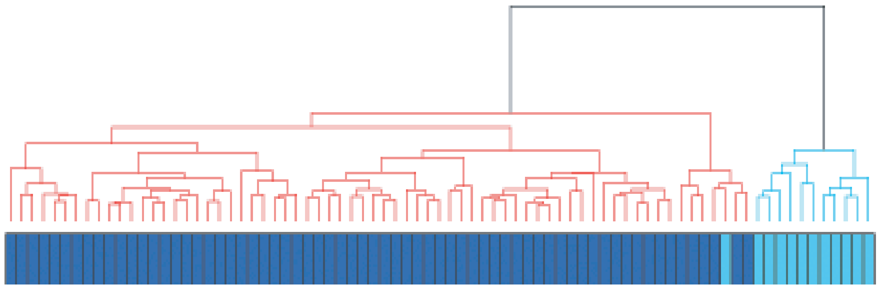

Jechlinger et al, 2003
Epithelial cells before and after
TGFbeta-induced EMT
χ^2 p-value: 6.9E-14
Bonferroni: 8.9E-13

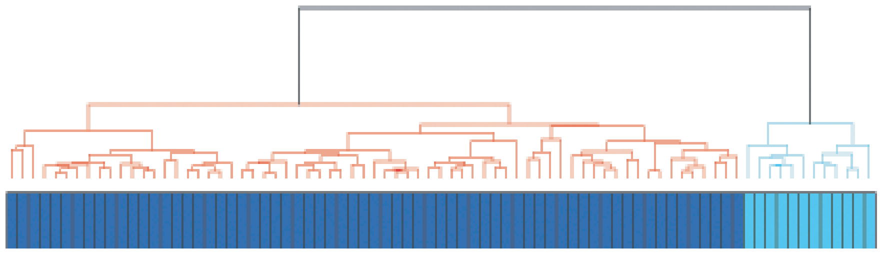

Raouf et al, 2008
Luminal (MUC1+ vs
bipotent (CD49f+, CD10+) cells
χ^2 p-value: 0.001
Bonferroni: 0.013

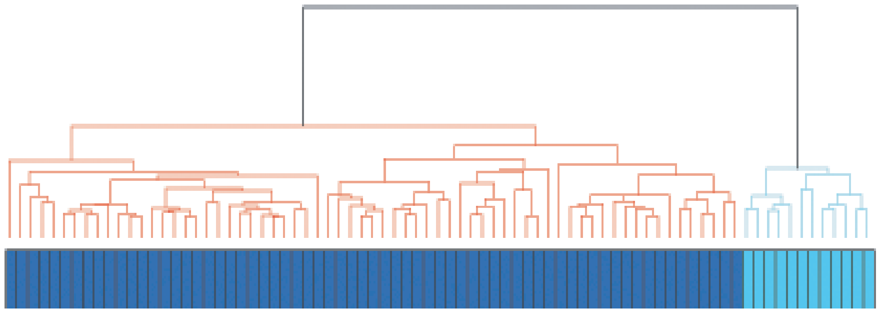

Raouf et al, 2008
Myoepithelial (CD10+) vs
bipotent (CD49+, CD10) cells
χ^2 p-value: 2.4E-5
Bonferroni: 3.1E-4

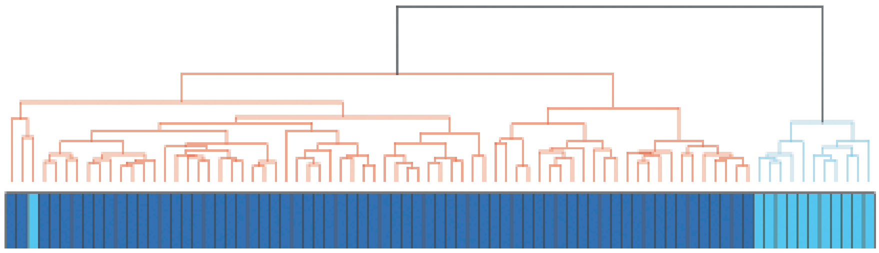

Liu et al, 2007
Stem-cell like (CD44+) vs
luminal (CD10+) cells
χ^2 p-value: 1.3E-05
Bonferroni: 1.7E-4

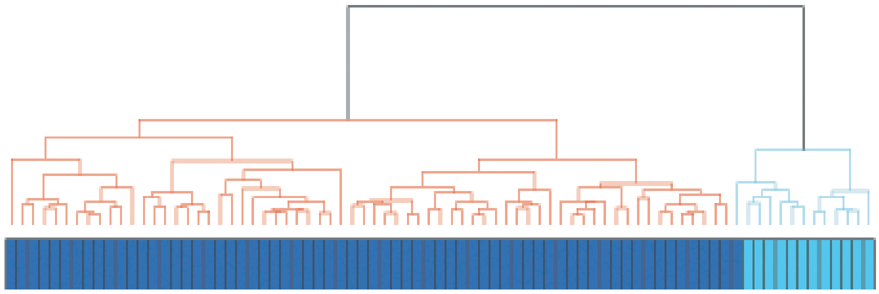

Jones et al, 2004
myoepithelial (MUC1+) vs
luminal (CD10+) cells
χ^2 p-value: 0.06
Bonferroni: 0.78

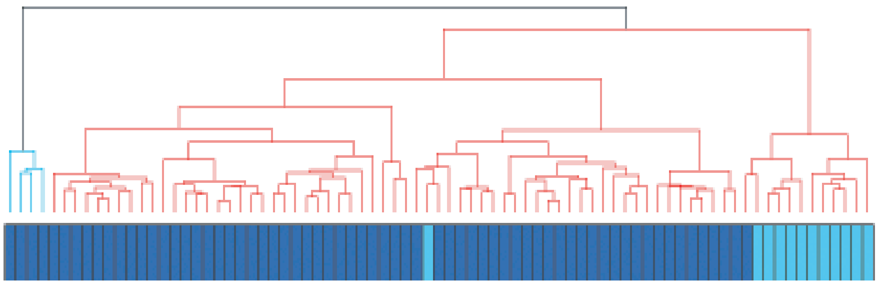

Chen et al, 2008
IDC-like normal cells vs
other normal cells
χ^2 p-value: 1.3E-9
Bonferroni: 1.7E-8

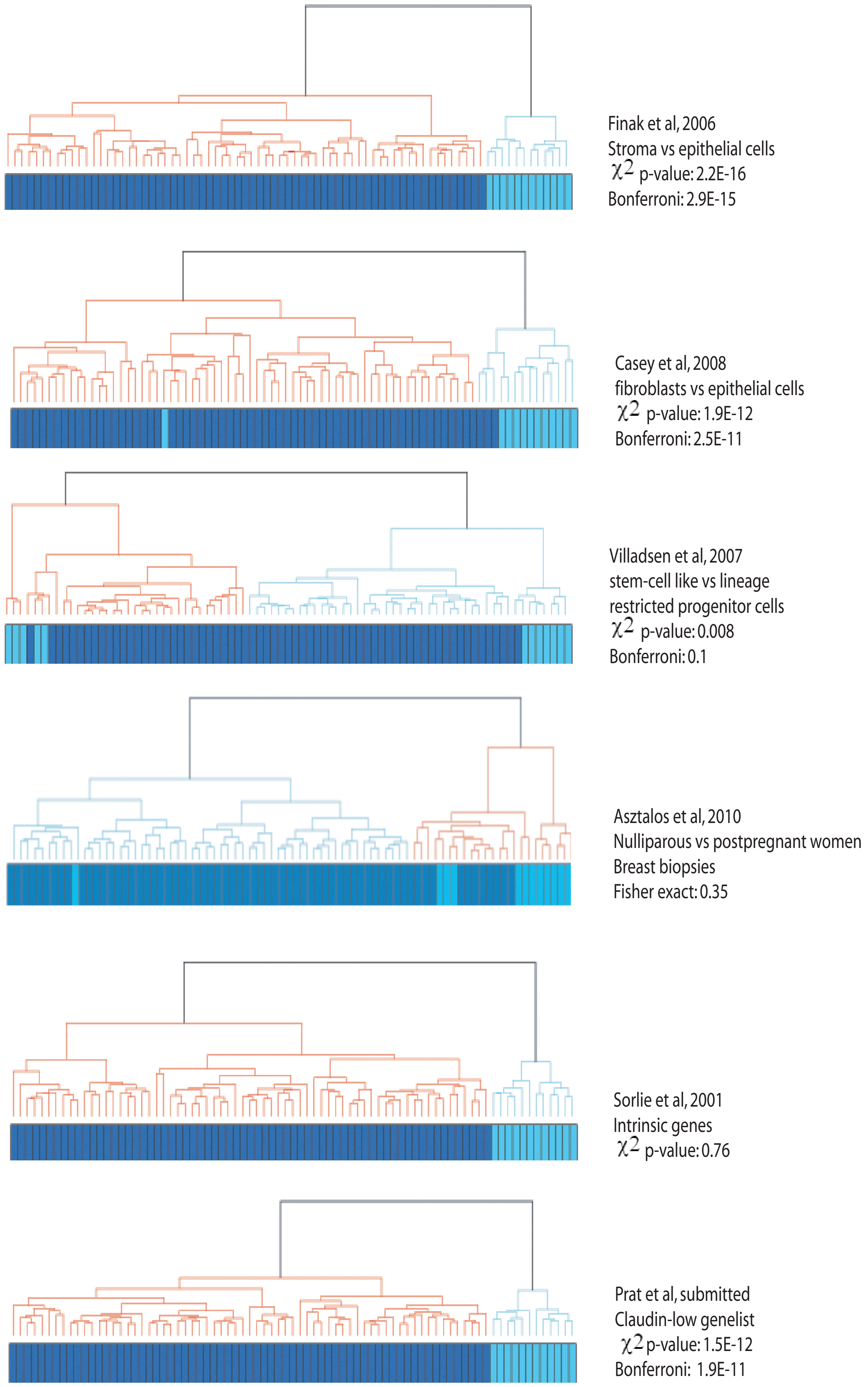

Hierarchical clustering of gene expression from 79 samples from breasts of healthy women. The samples are clustered based on gene lists from the literature, describing different cell types. The two last panels are clustered based on gene lists used to identify breast cancer subtypes. Cluster 1-samples are marked light blue and cluster 2-samples dark blue. The dendrogram colors represent the two main clusters in the clustering performed based on the gene list in question.

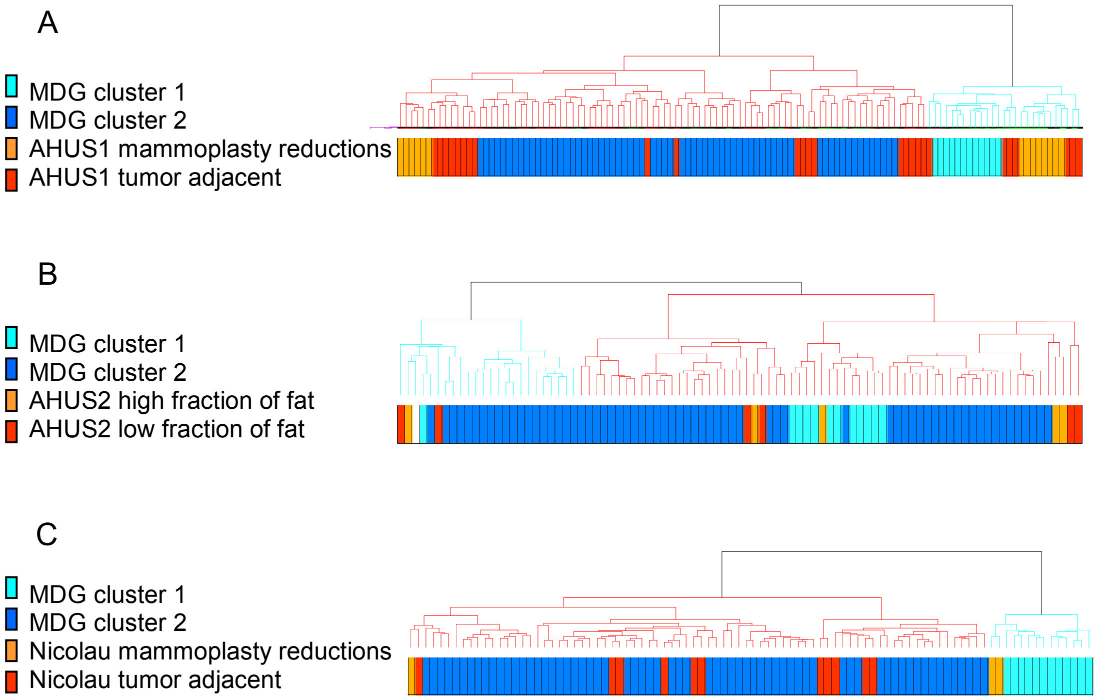

Figure S2 Biopsies from healthy women (MDG) clustered with two unpublished datasets from Akershus University Hospital (AHUS) A) AHUS1 with breast biopsies from mammoplasty reductions (yellow) and tumor adjacent (red) tissue and B) AHUS2 with breast biopsies containing different known proportions of fat tissue and C) a dataset previously published by Nicoalu et al [1] with breast biopsies from mammoplasty reductions (yellow) and tumor adjacent (red) breast tissue. In all cases, the two datasets are merged by use of Distance Weighted Discrimination (DWD). This resulted in datasets with A) 8520 genes, B) 10078 and C) 3555 genes. Hierarchical clustering with Eucledian distance and Ward linkage was performed as described. Ward linkage was performed as described in Materials and methods.

Reference list

1. Nicolau M, Tibshirani R, Borresen-Dale AL, Jeffrey SS: Disease-specific genomic analysis: identifying the signature of pathologic biology. Bioinformatics 2007; 23**:** 957-965.

Figure S3

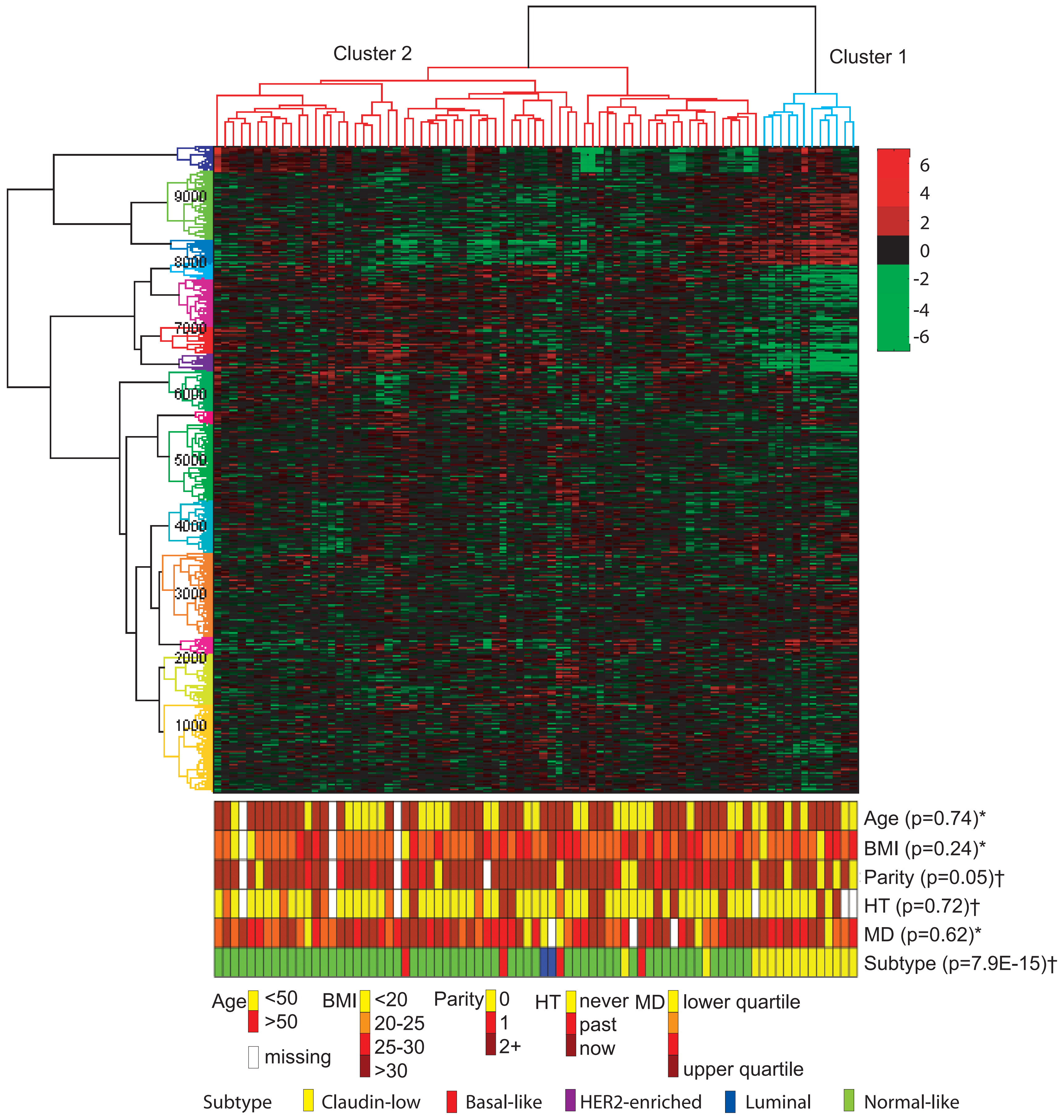

Figure S3 Unsupervised hierarchical clustering of 79 samples from healthy individuals as shown in Figure 1. Phenotypes with tests for significant difference in values between cluster 1 (blue) and cluster 2 (red). Continuous variables are categorized for the illustration, but significance tested as continuous variables. P-values from two-sided t-tests assuming equal variance for continuous variables (*) and chi-squared tests (**) for categorical variables are given. The numbers along the y-axis denotes the number of genes. The claudin-low subtype is found by use of a predictor as described in the Methods section. PAM50 is used to estimate subtypes for the non-claudin-low samples.
Age= Age at time of inclusion. BMI: Body mass index. HT: Use of hormone therapy. MD: Mammographic density.
